# Development and characterization of a multimeric recombinant protein using the spike protein receptor binding domain as an antigen to induce SARS‐CoV‐2 neutralization

**DOI:** 10.1002/iid3.1353

**Published:** 2024-07-26

**Authors:** Veronica A. de Lima, João P. S. Nunes, Daniela S. Rosa, Rodrigo Ferreira, Maria L. V. Oliva, Robert Andreata‐Santos, Marcia Duarte‐Barbosa, Luiz M. R. Janini, Juliana T. Maricato, Milena A. Akamatsu, Paulo L. Ho, Sergio Schenkman

**Affiliations:** ^1^ Department of Microbiology, Immunology and Parasitology Universidade Federal de São Paulo São Paulo São Paulo Brazil; ^2^ Department of Biochemistry, Escola Paulista de Medicina Universidade Federal de São Paulo São Paulo São Paulo Brazil; ^3^ Núcleo de Produção de Vacinas Bacterianas, Centro BioIndustrial, Instituto Butantan São Paulo São Paulo Brazil

**Keywords:** COVID ‐19, neutralizing antibody, protein S, receptor‐binding domain, SARS‐CoV‐2

## Abstract

**Background:**

SARS‐CoV2 virus, responsible for the COVID‐19 pandemic, has four structural proteins and 16 nonstructural proteins. S‐protein is one of the structural proteins exposed on the virus surface and is the main target for producing neutralizing antibodies and vaccines. The S‐protein forms a trimer that can bind the angiotensin‐converting enzyme 2 (ACE2) through its receptor binding domain (RBD) for cell entry.

**Aims:**

The goal of this study was to express in HEK293 cells a new RBD recombinant protein in a constitutive and stable manner in order to use it as an alternative immunogen and diagnostic tool for COVID‐19.

**Materials & Methods:**

The protein was designed to contain an immunoglobulin signal sequence, an explanded C‐terminal section of the RBD, a region responsible for the bacteriophage T4 trimerization inducer, and six histidines in the pCDNA‐3.1 plasmid. Following transformation, the cells were selected with geneticin‐G418 and purified from serum‐fre culture supernatants using Ni2+‐agarand size exclusion chromatography. The protein was structurally identified by cross‐linking and circular dichroism experiments, and utilized to immunize mice in conjuction with AS03 or alum adjuvants. The mice sera were examined for antibody recognition, receptor‐binding inhibition, and virus neutralization, while spleens were evaluated for γ‐interferon production in the presence of RBD.

**Results:**

The protein released in the culture supernatant of cells, and exhibited a molecular mass of 135 kDa with a secondary structure like the monomeric and trimeric RBD. After purification, it formed a multimeric structure comprising trimers and hexamers, which were able to bind the ACE2 receptor. It generated high antibody titers in mice when combined with AS03 adjuvant (up to 1:50,000). The sera were capable of inhibiting binding of biotin‐labeled ACE2 to the virus S1 subunit and could neutralize the entry of the Wuhan virus strain into cells at dilutions up to 1:2000. It produced specific IFN‐γ producing cells in immunized mouse splenocytes.

**Discussion:**

Our data describe a new RBD containing protein, forming trimers and hexamers, which are able to induce a protective humoral and cellular response against SARS‐CoV2.

**Conclusion:**

These results add a new arsenal to combat COVID‐19, as an alternative immunogen or antigen for diagnosis.

## Graphical abstract

We generated a new trimeric recombinant continuously expressed in HEK‐293 cells corresponding to the receptor binding domain and the C‐terminus of S1 protein SARS‐Cov2. It was able to generate neutralizing antibodies and suitable for diagnostic procedures of Covid‐19.

## 1 INTRODUCTION

In December 2019, patients in Wuhan, the capital of China's Hubei Province, experienced symptoms of severe influenza, mainly affecting the respiratory tract, which were initially reported to the Wuhan Municipal Health Department as unexplained cases of pneumonia. At the same time, the Wuhan Center for Disease Control and Prevention discovered that these patients were associated with a seafood market.^1^ Sequencing of samples isolated from the patients confirmed that the pathogen was a new type of coronavirus. As cases increased worldwide, human‐to‐human transmission was identified as the main cause of transmission. Epidemiological studies reported the occurrence of infection in people who did not have access to the seafood market in Wuhan.^2^ On February 11, 2020, the World Health Organization designated the disease caused by the SARS‐CoV‐2 virus as Coronavirus Disease‐2019 (COVID‐19), and on March 20, 2020, it designated COVID‐19 as a pandemic. By November 2023, there were more than 772 million confirmed cases and more than 6.6 million deaths worldwide (https://www.who.int/emergencies/diseases/novel-coronavirus-2019/situation-reports).

Coronaviruses belong to the Coronaviridae family of the subfamily Coronavirinae, which includes four genera: Alphacoronavirus, Betacoronavirus, Gammacoronavirus, and Deltacoronavirus.^3^ There are seven species of human coronaviruses (HCoV), four of which circulate and usually cause mild infections; HCoV‐29E, HCoV‐OC43, HCoV‐NL63, and HCoV‐HKU1,^4^ and three that cause more severe respiratory symptoms: SARS‐CoV, MERS‐CoV, and SARS‐CoV‐2. SARS‐CoV‐2 has approximately 80% similarity to SARS‐CoV and 51.8% similarity to MERS‐CoV,^5^ but the closest genetic identity of SARS‐CoV‐2 is SARS‐RaTG13 bat CoV with approximately 96.2% compatibility.^6^ The evolutionary ability of coronaviruses to evolve and to diverge has been recognized, which implicates them in the development of new diagnostic processes, as a large number of new coronavirus genomes are appearing continuously.^7^

SARS‐CoV‐2 is a betacoronavirus whose genome is a positive single‐stranded RNA that varies in size from 27 to 32 kbases. It contains four structural proteins and 16 nonstructural proteins.^3^ These structural proteins are Spike protein (S), a transmembrane homotrimeric glycoprotein formed by two subunits, the S1 subunits, and responsible for viral entry into the host cell through its receptor‐binding domain (RBD), which binds to angiotensin‐converting enzyme 2 (ACE2) on the host cell. The S2 subunit is responsible for viral and host cell membrane fusion.^8^

Protein S is the most important target for vaccine development because it generates neutralizing antibodies, which prevents its interaction with the cell receptors.^9^ As a result, several studies have been conducted using the entire S protein with minor modifications as a vaccine candidate,^10^ and several protective monoclonal antibodies have been obtained.^11^ However, the generation of neutralizing antibodies requires the protein to be in a state before fusion processing occurring during virus cell fusion.^12^, ^13^, ^14^ Immunization with the RBD, which is part of the S1 subunit mediates SARS‐CoV2 entry into the host cell and can also produce protective antibodies.^15^ The RBD contains an extended insert, the receptor binding motif, with amino acid residues that bind to the N‐terminus of ACE2.^16^ Multimeric RBDs have been shown to be more efficient at generating neutralizing antibodies because they can increase B‐cell activation,^17^ enhance neutralizing antibody responses, and prevent the formation of low‐affinity antibodies.^18^ Therefore, several studies have shown that trimeric RBD induces higher titers of neutralizing antibodies than monomeric RBD.^19^ Immunization of *Rhesus* macaques with various forms of protein S, for example, resulted in protective humoral and cellular responses, in addition to neutralizing antibody titers.^20^

There are currently more than 400 preparations in clinical development and preclinical development (https://www.who.int/publications/m/item/draft-landscape-of-covid-19-candidate-vaccines). The main available vaccines are from Moderna (mRNA‐1273) and Pfizer/BioNtech (BNT162b2) that use messenger RNAs, which encodes glycoprotein S, producing high titers of neutralizing antibodies with efficacy of 94% to 95%.^21^, ^22^, ^23^, ^24^ The vaccine ChAdOx1 nCoV‐19 (AZD1222) consists of an adenoviral vector of chimpanzees with replication defects that contains as antigen the glycoprotein gene S. This vaccine showed an efficacy of 70%.^25^ The Ad26.COV2.S vaccine produced by Johnson & Johnson is made of a human adenovirus serotype 26 (rAd26) that expresses the protein S.^26^ It was effective after a single dose with a high humoral response.^27^ Sputnik V, another vaccine that uses the human adenoviral vector rAd26 and rAd5 has been shown to induce strong humoral and cellular responses after two doses.^28^ The entire virus was also used in the production of the CoronaVac vaccine, which consists d of the inactivated SARS‐CoV‐2 virus^29^ and was able to elicit a strong immune response.^30^

After universal immunization, there was a considerable decrease in incidence, but the surge of new variants, such as lineage B.1.1.7, raised concerns regarding the efficacy of existing vaccines.^31^ These variants were classified as variants of interest (VOI), variants of concern (VOC), and variants under monitoring (VUM).^32^ With the emergence of the Omicron variant, which contains a higher number of mutations in the RBD compared to the other variants, it poses a threat to the effectiveness of the existing vaccines. Omicron has evolved significantly with challenging sub‐lineages, resulting in an approximately eightfold reduction in the neutralization titer.^33^

Furthermore, it is possible to detect SARS‐COV2 in fecal specimens from patients with Covid's pneumonia that present negative results in multiple reverse‐transcription polymerase chain reaction testing on oropharyngeal and nasopharyngeal swab specimens.^34^ The virus could be carried by bacteria colonizing the gut microbiota,^35^ which demonstrate the requirements for improved diagnosis for detection of infected patients and possible the use of alternative ways of infection control.^36^, ^37^

Despite the fact that numerous studies have generated proteins with the antigenic properties of the SARS‐CoV‐2 RBD, our group has designed an antigen that contained, in addition to the RBD domain, a C‐terminal portion of the S1 protein linked to the trimerization region of bacteriophage T4 in the C‐terminus of the protein to generate a robust and stable antigen expression for diagnostics, immunization, and neutralization assays. According to recent structural studies, this added C‐terminal might fold the trimer in a form comparable to the S1 protein, which would produce an improved antigen for immunization and diagnostic tests. In addition, unlike many previous studies, we generated cells stably expressing the protein, allowing the simpler production and purification of a trimeric form of the RBD antigen, which can be further adapted for large‐scale production.

## 2 MATERIALS AND METHODS

All procedures, reagents, and methods were performed according to compliance rules.

### 2.1 Animals

Female BALB/c mice aged 6–8 weeks were obtained from the Centre for the Development of Experimental Models for Medicine and Biology, CEDEME–Universidade Federal de São Paulo (UNIFESP). Animals were maintained under standard lighting conditions (12 h of light and 12 h of darkness) at a controlled temperature (25 ± 2°C) with food and water ad libitum in the vivarium of the Department of Microbiology at the Escola Paulista de Medicina of UNIFESP. For euthanasia, animals received a lethal dose of xylazine and ketamine intraperitoneally in accordance with ethics committee regulations. All procedures, in this work were approved by the Institutional Animal Care and Use Committee (IACUC) of UNIFESP (Case No. 8507290721). All used animals were included in the reported results.

### 2.2 Plasmid and recombinant production

The pcDNA 3.1 (+) plasmid containing the RBD sequence of the protein (encompassing the region between amino acids 331 and 589 of the SARS‐CoV‐2 S protein (MT350282) of the Wuhan strain was synthesized by GeneScript after codon optimization for expression in mammalian cells. The insert DNA sequence is shown in Supporting Information S1: Figure S1. The plasmid was grown in *Escherichia coli* Mach1 (provided by the Structural Genomic Consortia; SGC Campinas) in Luria‐Bertani medium containing 100 µg/mL ampicillin (Sigma‐Aldrich). Plasmid DNA was prepared using the Midi‐Prep kit (Sigma‐Aldrich), and preparations were analyzed after digestion with endonucleases by electrophoresis in 0.8% agarose gel in TAE buffer (40 mM Tris‐HCl, 0.3 mM acetic acid, and 2 mM EDTA).

### 2.3 Cell culture

Human cells (HEK293) (Human embryonic kidney 293, ATCC CRL‐1573), kindly provided by Dr. João Bosco Pesquero, UNIFESP, were cultured with RPMI‐1640 (Thermo Fisher Scientific), 50 U/mL penicillin, and 50 µg/mL streptomycin, supplemented with 10% fetal bovine serum (FBS) (InvitroCell), and maintained at 37°C and 5% CO_2_. For transfections, 2 × 10^4^ HEK293 cells were added to each well of a six‐well plate, and after 24 h at 37°C, cells were transfected with the linearized plasmid DNA‐lipofectamine LTX complex (Thermo Fisher Scientific) according to the manufacturer's recommendations. Briefly, 0.5 µg of plasmid pcDNA 3.1/RBD was linearized with the BamHI enzyme (Thermo Fisher Scientific), precipitated, and washed with 70% ethanol. The DNA was resuspended in 285 µL of Opti‐MEM (Thermo Fisher Scientific). Then, 15 µL of Lipofectamine LTX (Thermo Fisher Scientific) was added and the solution was incubated for 20 min at room temperature to form the DNA‐lipofectamine complex. The cell monolayers were washed once with Opti‐MEM, and 0.5 mL of Opti‐MEM, containing the plasmid, was added to each well. Cells were incubated at 37°C, and after 24 h, the medium was replaced, and selection was initiated with 0.5 mg/mL Geneticin‐G418 (InvivoGen). Plasmid‐free transfected cells were used as a kill control. To confirm secretion of the recombinant protein RBD, cells were cultured with RPMI and 10% FBS until they reached 90% confluence. After reaching confluence, cells were cultured with medium without FBS (OptiPRO SFM, 12309019; Thermo Fisher Scientific), and the supernatant was collected every 4 days during 5 weeks.

### 2.4 Western blot analysis and immunofluorescence

For western blot analysis assays, samples were boiled for 5 min with sample buffer (50 mM Tris‐HCl, pH 6.8, 0.02% bromophenol blue, 2% SDS, and 20% glycerol with or without 10% β‐mercaptoethanol), applied to a 10% sodium dodecyl sulfate–polyacrylamide gel electrophoresis (SDS‐PAGE) gel, and transferred to 0.45‐µm nitrocellulose membranes (Bio‐Rad), using standard procedures. The membranes were blocked with 5% bovine serum albumin (BSA) or 5% skim milk powder in 0.15 M NaCl, 10 mM Tris‐HCl, pH 7.4 (TBS), for 1 h. The membranes were then incubated for 1 h with serum from horses immunized with Newcastle virus expressing spike antigen^38^ at a dilution of 1:10.000 in blocking solution or with serum from mice immunized with a recombinant RBD protein produced in bacteria (kindly provided by Dr. Santuza Teixeira from the Federal University of Minas Gerais), or also with the sera from mice immunized with the recombinant protein produced in this work. After washing the membranes with TBS with 0.05% Tween 20 (TBS‐T), bound equine antibodies were detected by incubation for 1 h with protein A conjugated to peroxidase (GE, 1:2000) and visualized with chemiluminescence reagent (ECL; Merck). Images were obtained with an Odyssey Li‐Cor instrument. Mouse antibodies were detected by using IRDye800 mouse anti‐IgG (Li‐Cor, 1:10.000) and observed with the same system.

For immunofluorescence, transfected cells (2 × 10^4^) were seeded onto 13‐mm glass coverslips in a 24‐well plate, and after 24 h, they were fixed with 4% p‐formaldehyde in phosphate‐buffered saline (PBS) (PFA), for 20 min on ice. Fixed cells were washed three times with PBS, permeabilized with 0.05% Triton X100 in PBS for 2 min, washed again in PBS, and maintained for 30 min with 3% BSA in PBS before incubation with the indicated serum. Bound antibodies were detected by adding Alexa488 conjugated to anti‐mouse IgG (1:10,000; Thermo Fisher Scientific) and observation under an Olympus BX61 fluorescence microscope.

### 2.5 Expression and purification of the recombinant protein

For protein production, aliquots of cells frozen in liquid nitrogen were grown in 75 cm^2^ culture flasks in RPMI‐1640 medium containing 10% FBS and 0.5 mg/mL Geneticin G418 until confluence was reached. Cells were trypsinized and seeded in four 150 cm^2^ flasks containing the same culture medium. After reaching 80% confluence, the medium was replaced by 25 mL of medium without geneticin G418 and FBS in OptiPro SFM (1230919; Thermo Fisher Scientific). The medium was collected and replaced after 4 days, and protein expression in the supernatant was monitored by western blot analysis. Culture supernatants that contained detectable recombinant protein were collected and stored at 4°C until purification.

Protein purification was performed by loading the culture supernatant at 1 mL/min into a Ni‐Sepharose HisTrap FF™ column (GE) pre‐equilibrated with 40 mM imidazole, 20 mM sodium phosphate, and 0.5 M NaCl, pH 7.4. In each batch, 400 mL of supernatant were used. After one passage, the column was washed with equilibration buffer, and the protein was eluted with 15 mL of 0.5 M imidazole, 0.5 M NaCl, and 20 mM sodium phosphate, pH 7.4, in three fractions of 5 mL. These fractions were concentrated by centrifugation on Centricon Plus‐20 5000 filters (Merck‐Millipore), and protein levels were determined by micro‐BCA assays (Thermo Fisher Scientific).

The concentrated samples (0.5 mL) were chromatographed on a Superdex 200 column (30 × 1 cm; Cytiva) equilibrated in TBS at a flow rate of 0.4 mL/min. Fractions of 0.25 mL were collected using an Akta purifier chromatographer (GE). For determination of molecular mass, the column was calibrated with BSA (66 and 132 kDa), carbonic anhydrase (30 kDa), and β‐amylase (200 kDa) (Sigma‐Aldrich).

### 2.6 Structural analysis

Circular dichroism (CD) measurements were performed using a Chirascan Plus spectrometer (Applied Photophysics) at 25°C; optical path of 0.1 cm, in quartz cuvettes. CD data were obtained in the range of 200–250 nm, with a 1 nm step, and a 1 nm window. Protein concentrations used were 0.345 mg/mL (2.5 µM), and spectra were obtained from the average of 8 scans. Spectra were converted to molar ellipticity (*θ*) using the equation below for secondary structure analysis, which was computed using CDPro software.^39^

In cross‐linking experiments, the purified recombinant protein in 25 mM sodium phosphate pH 7.4 was incubated for 1 h in 2 mM or 5 mM of suberic acid bis(3‐sulfo‐N‐hydroxysuccinimide ester) sodium salt (Sigma‐Aldrich). To stop the reaction, 25 mM of Tris‐HCl pH 7.4 was added, and the mixture was incubated for another 10 min. The samples were analyzed by western blot analysis as described above.

### 2.7 Immunization and immunoassays

Two series of immunizations were performed, always in the morning. In the first series, five mice were given 100 µg of multimeric RBD each that had been purified on a Ni‐agarose column with the same amount of aluminum hydroxide (Inject Alum; Thermo Fisher Scientific). The controls consisted of five mice immunized with PBS mixed with alum. In the second series, the mice were divided into three groups, each with five mice. Two experimental groups received 100 µg of highly purified RBD‐Multi, diluted in PBS with the same volume of aluminum hydroxide (Inject Alum; Thermo Fisher Scientific) or diluted (1:1 v/v) in the presence of AS03 adjuvant (InvivoGen). The third group only received PBS. In both series, animals were immunized intramuscularly in the hind paw with four 0.05 mL doses (Prime, Boost I, Booster II, and Booster III), 2 weeks apart. Blood was drawn from the tail vein (10 µL) 15 days after each dose to check antibody titers. Animals were anesthetized 15 days after the last dose, and blood was collected via cardiac puncture. In the second series the spleen was also removed for the T‐cell ELISpot assay (see below).

For enzyme‐linked immunosorbent assay (ELISA) assays, a white high‐binding polystyrene plate (Sarstedt) was used. It was sensitized by the addition of 200 ng of the purified multimeric RBD protein diluted in 50 µL of 0.1 M sodium bicarbonate pH 8.5 per well, and incubated overnight at 4°C. The wells were washed twice with PBS‐Tween 0.05% (PBS‐T), and incubated with 5% skim milk powder in PBS for 1 h and then with the sera diluted in the same solution for 1 h, at 37°C. The wells were washed again in PBS‐T and incubated with 100 µL of 1/10,000 diluted goat anti‐mouse IgG peroxidase (Thermo Fisher Scientific). After 1 h, the wells were washed, and binding was detected by the addition of the ECL chemiluminescent reagent (Merck‐Millipore). The light generated was measured by the Spectramax M3 (Molecular Devices) plate reader at a wavelength of 700 nm.

### 2.8 Competitive enzymatic immunoassay

The presence of neutralizing antibodies was determined using the enzyme immunoassay adapted from NeutraLISA (EUROIMMUN, Brazil, kindly donated by Dr. Michael Soane). The kit contained a plate sensitized with the SARSCoV2 S1 protein. One hundred µL of a mixture of the test serum (final dilution 1/5) and the kit ACE2‐biotin solution were added. After 1 h at 37°C, the wells were washed with the kit wash solution and incubated with the kit streptavidin‐peroxidase solution for an additional 30 min at room temperature. The wells were washed again, and binding was detected by incubation with the chromogen contained in the kit for 15 min after the reaction was terminated by the addition of the kit stop solution. Absorbance values were then measured at 450 nm using the SpectraMax M3 plate reader. The same method was adapted by coating 100 µL of high‐binding transparent polystyrene plates with 50 ng of the recombinant RBD protein, as described in the ELISA assay. The next day, the wells were washed with the solution provided in the kit, and the procedure was repeated as described above. To obtain the blank value, only the solution containing biotinylated ACE2 (EUROIMMUN) was used. Assay values were plotted as % inhibition using the following calculation: 100% − (sample value × 100/blank value) = % inhibition.

### 2.9 Virus neutralization test

Cytopathic effect SARS‐CoV‐2 virus neutralizing tests were performed to quantify neutralizing antibody titers as described earlier.^40^, ^41^, ^42^ For this, monolayers containing 5 × 10^4^ Vero cells (ATCC CCL‐81) were grown in 96‐well plates and exposed to Tissue Culture Infectious Dose (TCID_50_) of the Wuhan Strain (SARS‐CoV‐2/human/BRA/SP02/2020 strain ‐ M T126808.1), Delta strain (gisaid EPI_ISL_2965577), strain P1 (gisaid EPI_ISL_1060981), Omicron strain (hCoV‐19/Brazil/SP‐HIAE‐ID990/2021 gisaid EPI_ISL_6901961) previously incubated with 1:40–1:2560 test sera. After 72 h of incubation, all wells were evaluated by optical microscopy for the presence of cytopathic effects, characteristic of SARS‐CoV‐2. The absence of cytopathic effects in at least the 1:40 dilution sample was considered a positive result of neutralizing antibodies for SARS‐CoV‐2. All cytopathic virus neutralization test (CPE‐VNT) procedures were performed at biosafety level 3 in the laboratory of the Federal University of São Paulo, according to World Health Organization recommendations.

### 2.10 T cell ELISpot assay

To analyze the response of interferon‐γ (IFN‐γ) secreting cells, the ELISPOT Mouse IFN‐γ Set Kit (BD Biosciences) was used. Plates with polyvinylidene fluoride bottoms (MAIPS4510; Millipore) were coated overnight at 4°C with an anti‐IFN‐γ antibody (51‐2525KZ; BD). The next day, the plates were blocked with R10 (RPMI‐1640 media supplemented with 10% FBS, 2 mM l‐glutamine, 1 mM sodium pyruvate, 1% v/v non‐essential amino acids, 1% v/v penicillin/streptomycin, and 5 × 10^−5^ M 2‐mercaptoethanol (all from Thermo Fisher Scientific), for 2 h at room temperature. Plates were then washed with sterile PBS, and splenocytes were added at 5 × 10^5^ cells/well in R10 containing 30 U/mL of recombinant IL‐2 (ZODIAC). Cells were stimulated for 18 h at 37°C in 5% CO_2_ atmosphere with the RBD‐multiprotein at a concentration of 5 µg/mL. After washing with PBS containing 10% FBS, an antimouse IFN‐γ detection antibody (51‐2525KZ; BD) was added and incubation for 2 h. Then the plate was washed and avidin‐HRP in PBS and 10% FBS was added. After 1 h, the wells were washed with PBS and 10% FBS, and the AEC substrate was added for 20–40 min. The reaction was stopped by adding running water. After drying the plates at room temperature in the shade, the plate was read with the AID EliSpot Reader System (Autoimmun Diagnostika GMbH) to count the spot forming units per million splenocytes (SFU/10^6^ cells). Concanavalin at a concentration of 1.25 μg/mL was used as a positive control. Wells stimulated with the protein were subtracted from unstimulated wells.

### 2.11 Statistical analysis

In vivo experiments were performed with a sample size of five animals per group and analyzed in duplicates from each animal. Results were expressed as mean ± standard deviation. Statistical tests were analyzed using GraphPad Prism 9 software, with significant differences considered when *p* < .05 with analysis of variance (ANOVA) test with Tukey's multiple comparison or *t*‐test.

## 3 RESULTS

### 3.1 Expression of the recombinant protein RBD in HEK293 cells

**Figure 1:**
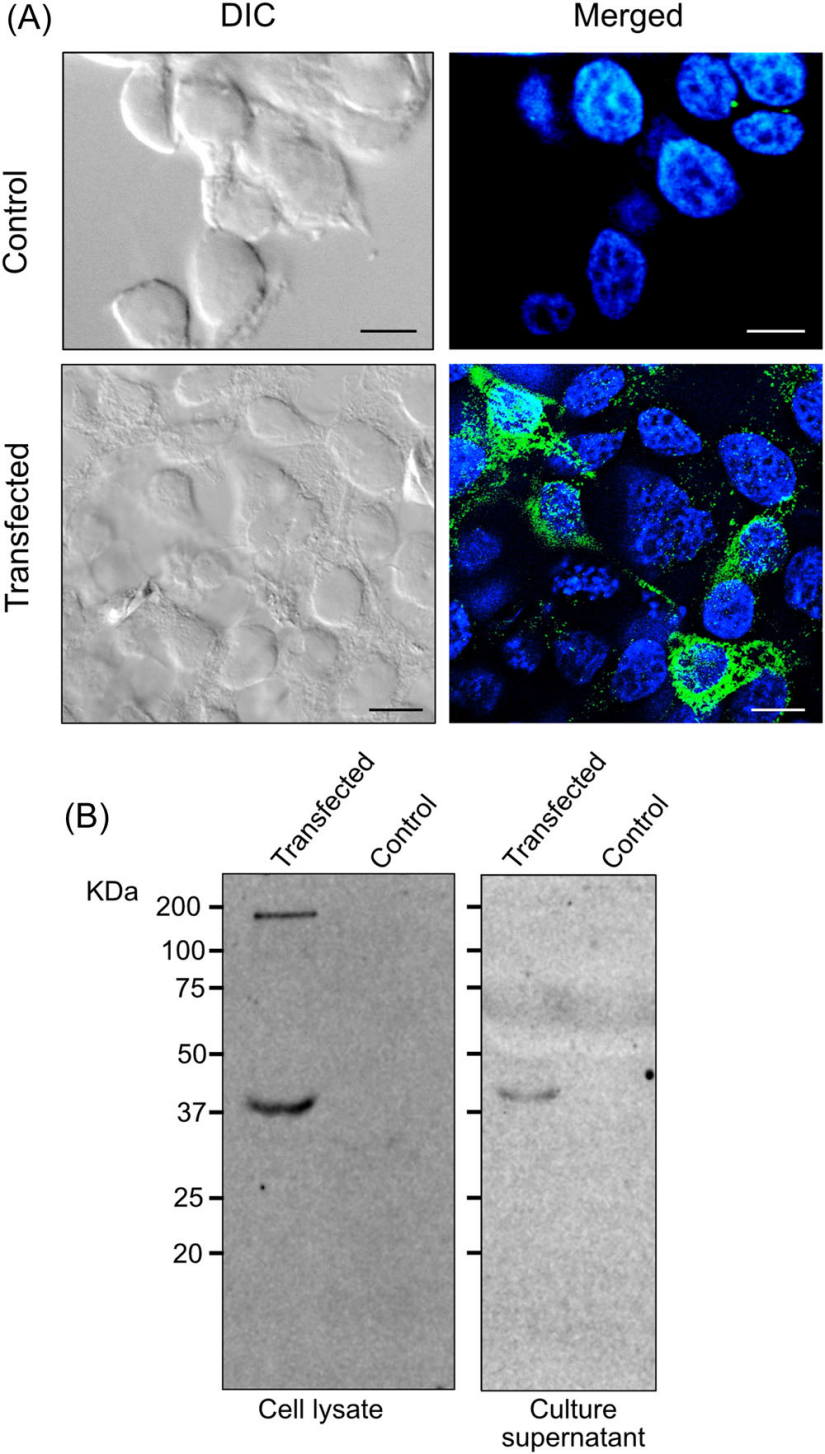
Receptor‐binding domain (RBD) is expressed and secreted by HEK293 cells. (A) Immunofluorescence of control and transfected HEK293 cells after G418‐selection cells using antimouse RBD antibodies (green) and 4′,6‐diamidino‐2‐phenylindole staining (blue). The images show DIC and merged fluorescent images. Bars = 5 µm. (B) Western blot analysis of cell lysates and culture supernatant collected after 24 h of culture of HEK293 cells containing the RBD plasmid and nontransfected cells revealed using horse serum anti‐RBD and peroxidase‐conjugated protein A. The size markers in kDa are shown on the left.

We employed the HEK293 cell line, which is widely used to produce recombinant proteins due to its machinery capable of performing much of the folding and posttranslational processing required to produce functional proteins derived from mammalian and nonmammalian DNA sequences.^43^ After transfecting the cells with the pcDNA 3.1/RBD plasmid and selecting cells resistant to geneticin G418, the cells were cloned. Before cloning, we used an immunofluorescence assay to detect reactivity with anti‐RBD antibodies (Figure 1A), which was later used to detect positive clones. One of these clones was used to test RBD protein expression by western blot analysis. As expected, HEK293 cells expressed and secreted the RBD in the supernatant (Figure 1B). The expected molecular mass for the recombinant protein is 28 kDa and a band with 37 kDa, suggests that it was glycosylated.

**Figure 2:**
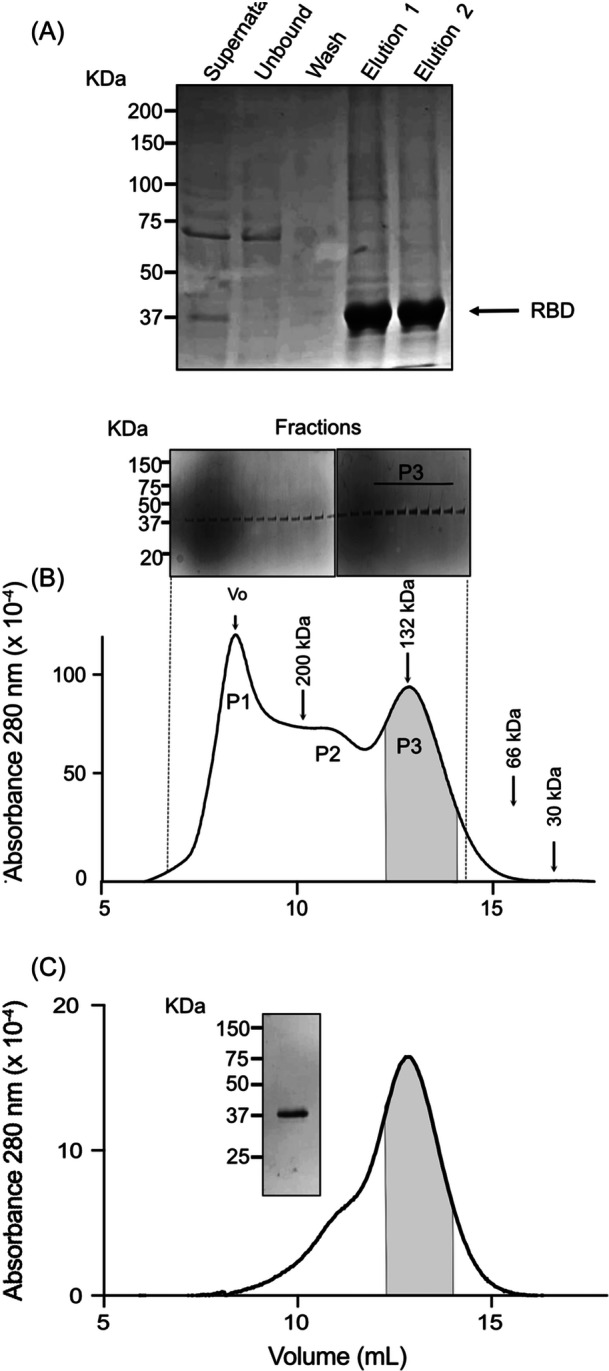
Receptor‐binding domain (RBD) purification steps. (A) Sodium dodecyl sulfate–polyacrylamide gel electrophoresis (SDS‐PAGE) gel stained with Coomassie blue R250 of 20 µL of samples of a total of 400 mL of culture supernatant applied in the Ni‐Sepharose column (supernatant). The fractions corresponding to unbound and washed eluates from the column and the two eluates (5 mL each) in buffers containing 0.5 M imidazole are indicated. (B) Size exclusion chromatography profile of the Superdex 200 column. The fractions eluted from the Ni‐Sepharose column were concentrated to 0.5 mL and applied to the column. Proteins were detected by absorbance at 280 nm. P1 corresponds to possible protein aggregates, P2 to protein forms with a mass higher than 200 kDa, and P3 to a mass of 125 kDa. The inset shows Coomassie bluer R250 stained SDS‐PAGE of the corresponding fractions eluted from the column. The arrows indicate the elution positions of the size markers (β‐amylase, 200 kDa; bovine serum albumin [BSA] dimer, 132 kDa; BSA monomer, 66 kDa; and carbonic anhydrase, 30 kDa). (C) Elution profile of concentrated samples of the first exclusion chromatography (gray area in B) in a new chromatography. The inset shows a typical SDS‐PAGE gel stained with Coomassie Blue R250 of a concentrated pool of fractions, as shown in the gray area. Size markers for SDS‐PAGE are indicated in kDa.

The RBD protein secreted in the supernatant of HEK293 cells was initially purified on a Ni‐Sepharose chromatography column, as it contained a histidine tag in the C‐terminus. Figure 2A shows an example of the results obtained after Ni‐Sepharose purification. The fractions eluted from the Ni‐Sepharose column with 0.5 M imidazole were concentrated and subjected to a second purification step on a Superdex 200 column. Some proteins with a mass greater than 250 kDa eluted in the column void, and two additional peaks were observed (Figure 2B). One eluted with a mass corresponding to 135 kDa, while the other had a mass of about 160 kDa. All of these samples contained the recombinant protein RBD, which migrated at 37 kDa on SDS‐PAGE (inset). The fractions eluting in the column's void volume contained less of the 37 kDa protein than the fractions eluting later. This occurs because protein aggregates enhance light absorption at 280 nm given the impression that more proteins are present in this fraction. The 135 kDa eluting fractions were pooled, concentrated, and re‐applied to the Superdex‐200 column. Subsequently, the protein was eluted as a homogeneous fraction with a mass of 135 kDa (Figure 2C). In summary, we obtained 2.8 mg of trimeric protein from 1 L of HEK293 cell supernatant. This yield is expected for a protein that forms aggregates and requires a further step of purification.^18^ The expression was obtained in monolayers growing continuously and could be increased by changing the culture flasks, media conditions or by generating cells in suspension.

### 3.2 The purified RBD forms multimeric structures

**Figure 3:**
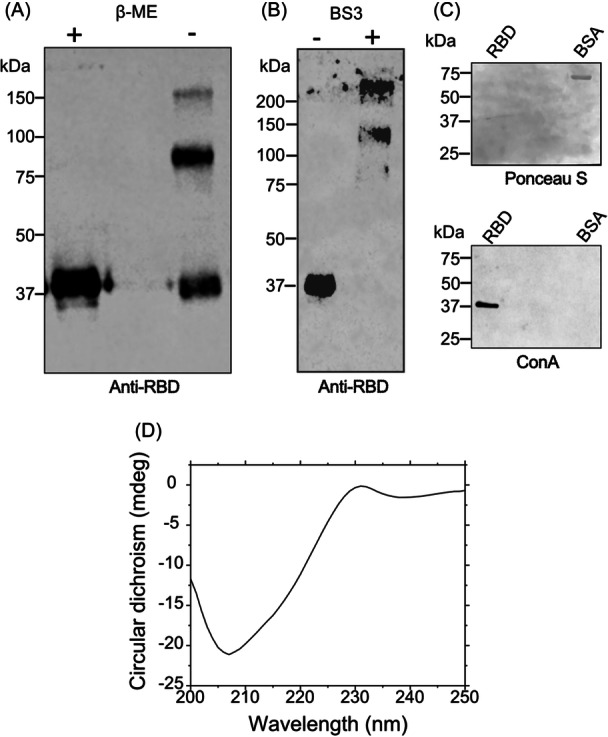
The recombinant receptor‐binding domain (RBD) forms a trimeric structure and presents a structured conformation. (A) The purified protein eluted from the second sizing column was concentrated and boiled in sample buffer in the presence (+) or absence (−) of β‐mercaptoethanol (βME) and submitted to western blot analysis using mouse anti‐RBD antibodies. (B) The purified RBD sample was treated in the absence (−) or presence (+) of the BS3 crosslinker for 1 h and submitted to western blot analysis using mouse anti‐RBD. (C) A nitrocellulose membrane obtained after transfer of a sodium dodecyl sulfate–polyacrylamide gel electrophoresis loaded with the recombinant RBD or bovine serum albumin and stained with Ponceau S (Top) or incubated with biotin‐Concanavalin A (ConA) and revealed with streptavidin‐peroxidase by ECL reaction. (D) Circular dichroism analysis of the purified RB at 0.3 mg/mL in TBS.

To ascertain whether the protein consisted of multiple subunits connected by sulfhydryl bridges, we conducted an SDS‐PAGE in the absence of the reducing agent, given that it eluted with an estimated 135 kDa from the size column. We observed that two‐thirds of the protein migrates with a mass of 75 kDa, whereas one‐third migrates with a mass of 37 kDa (Figure 3A). This implies that a dimer made up of two covalently bonded monomers via at least one disulfide bond and one monomer form was generated by a dimer consisting of two covalently linked monomers through at least one disulfide bond, and one monomer generated a trimeric structure. This is in line with the fact that each monomer in our construction has nine cysteines, of which eight form intramolecular disulfide bridges and one sulfhydryl remains free. A band with a higher mass could correspond to structures of larger size, consistent with the second peak in the elution profile of a band above 150 kDa in the Superdex‐200 column.

We treated the purified protein with BS3, a crosslinking reagent that links nearby free amino groups, to see if these structures occurred. We detected two major bands, one of 130 kDa and the other of roughly 260 kDa (Figure 3B), which correspond to three and six units of the original protein, respectively. We further established that RBD multimers contained glycosidic residues due to their reactivity with concanavalin A (Figure 3C) and had a folded structure, as shown by circular dichroism analysis (Figure 3D), with similar structures compared to monomeric RBD.^44^

### 3.3 The multimeric RBD induced high titers of antibodies in mice

**Figure 4:**
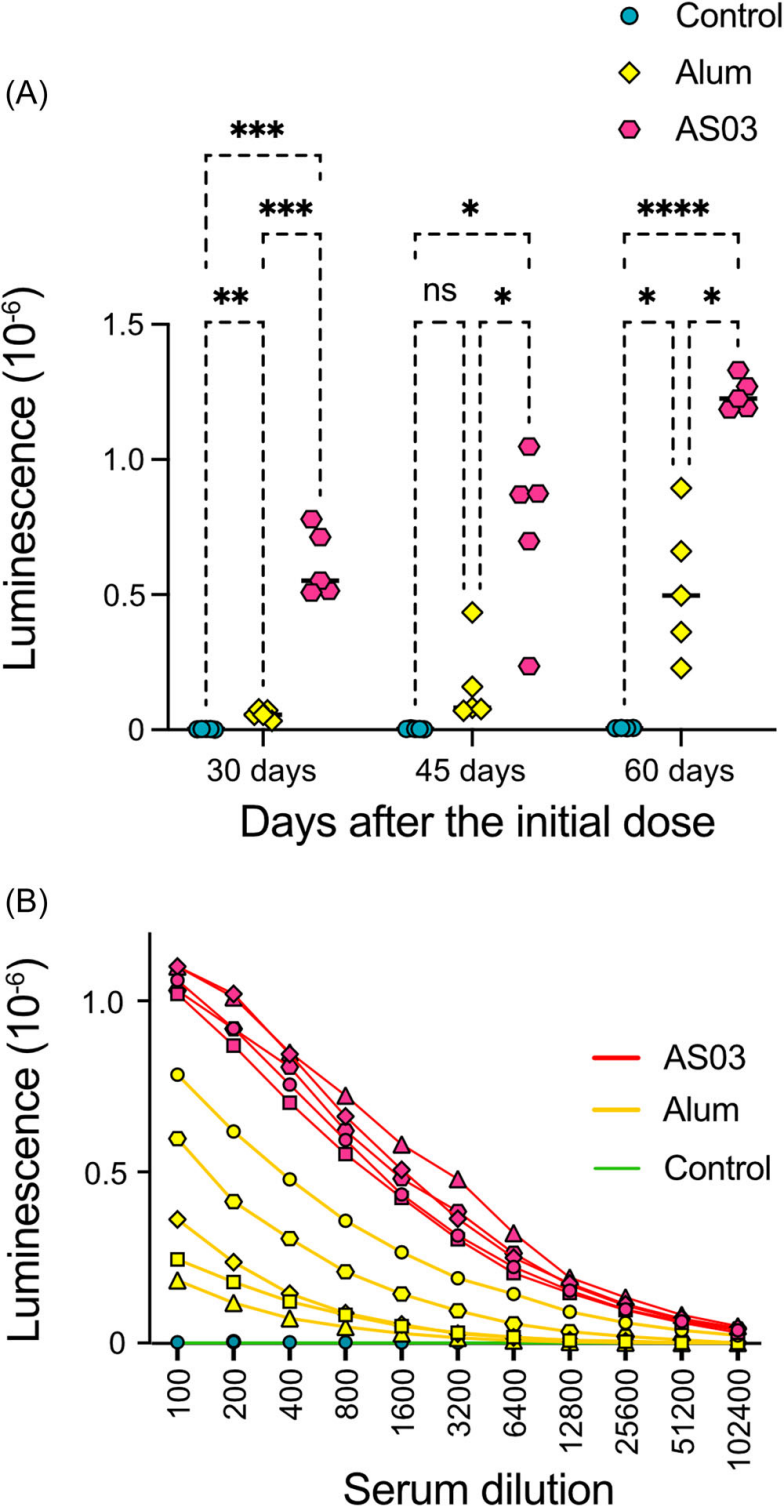
Enzyme‐linked immunosorbent assay (ELISA) assay used to evaluate the reactivity of sera to the multimeric receptor‐binding domain (RBD). (A) Purified multimeric RBD (200 ng) was used to coat an ELISA plate. Assays were performed in duplicate with sera diluted 1:100. Bound antibodies were detected with peroxidase‐coupled anti‐mouse IgG antibodies and visualized with ECL. Signals correspond to the mean of duplicates of sera obtained from mice immunized with PBS (control), mice immunized with RBD‐multi and Alum adjuvant (Alum), and mice immunized with RBD‐multi and AS03 adjuvant (AS03). The significance of the indicated differences was calculated using the two‐way analysis of variance with Tukey's multiple comparison test, where ****p < .0001, ***p < .001, **p < .01, *<.1, and ns, represent not significant. (B) Determination of the titer of pooled sera obtained after the third immunization in plates coated with the indicated amounts of RBD per well. Points represent the mean of duplicates for each dilution of sera incubated in PBS with 3% BSA.

To evaluate the immunogenicity of the multimeric RBD, mice were immunized intramuscularly at 2‐week intervals with four doses containing 100 µg of the purified recombinant RBD (second series) mixed with aluminum hydroxide or mixed with AS03. The control animals received only PBS. After each administration, 10 µL of blood was collected from each animal to analyze antibody production. There were no detectable levels of antibodies in serum diluted 1/100 after the first dose of the immunogen using a chemiluminescent ELISA assay with plates containing recombinant RBD (Figure 4A). After the second dose of antigen in AS03, a response was observed, which increased after the third injection. When the antigen was injected with alum, RBD antibodies did not appear until the third dose. Nonetheless, both adjuvants elicited a response 60 days after the initial immunization. The titers of the pooled antisera for each group of animals were subsequently determined using ELISA assays. For this experiment, 200 ng of the multimeric RBD was utilized to coat each well of the plates. Reactivity was observed at dilutions at 1:50.000 (adjuvanted with alum) and at 1:100.000 (adjuvanted with AS03) (Figure 4B). At a dilution of 1:100, the pre‐immune pool exhibited no reactivity.

### 3.4 Analysis of antibody neutralization by competition assay with ACE2

**Figure 5:**
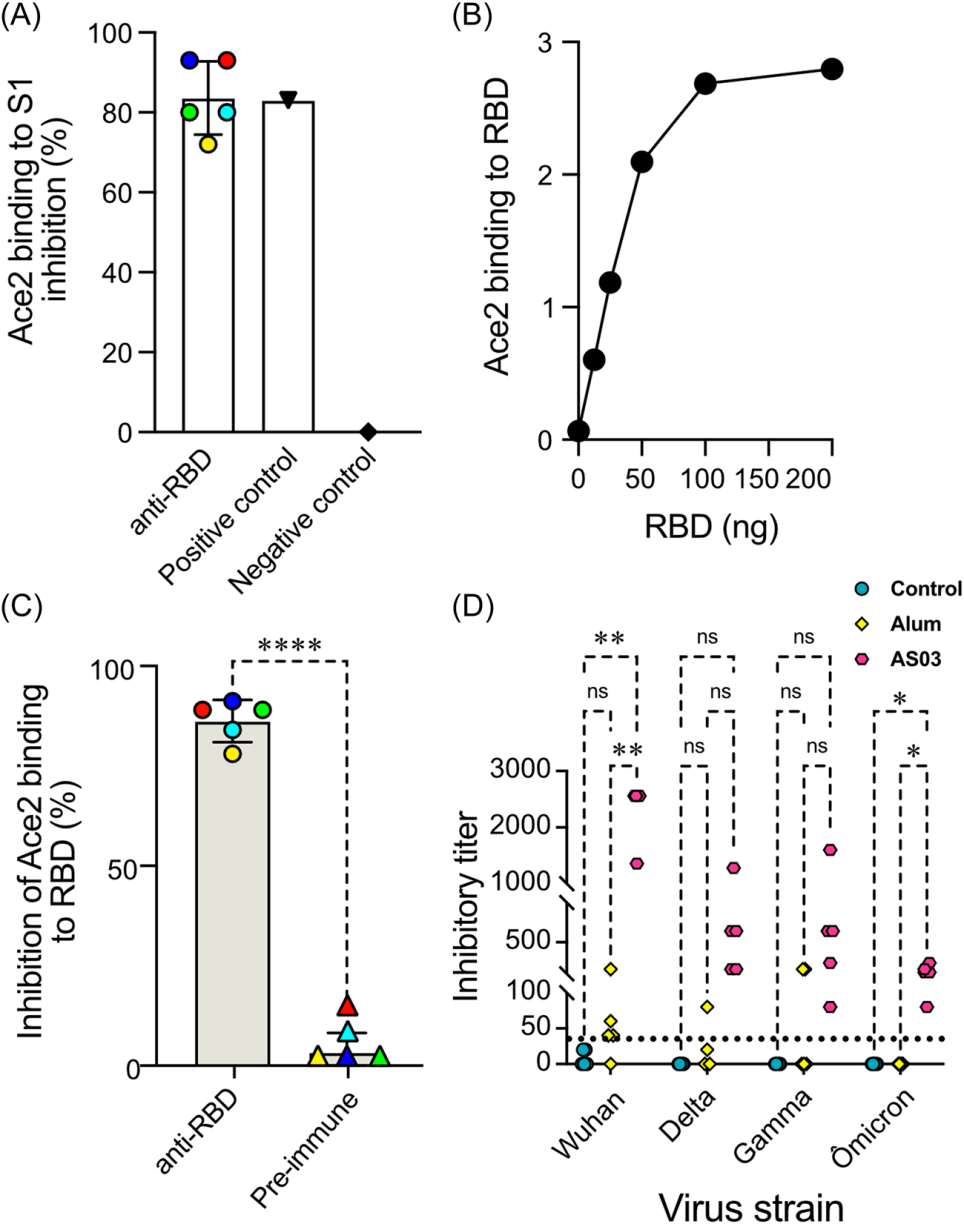
Mouse antibodies block ACE2 binding to the multimeric receptor‐binding domain (RBD) and prevent virus infection. (A) Competition assay of ACE2 binding to immobilized S1 protein. Wells were incubated with the ACE2‐biotin solution in the presence of the positive (+) and negative (−) controls provided with the kit and with mouse sera collected after the third immunization. (B) ACE2‐biotin protein binding to wells coated with the indicated amounts of recombinant RBD in 50 µL of 0.1 M sodium bicarbonate buffer, pH 8. After washes, the wells were incubated with streptavidin solution coupled to peroxidase and associated peroxidase measured by the immunogen detection system. (C) In vitro neutralization assay of ACE2‐biotin binding to wells coated with 50 ng of multimeric RBD in the presence of 1:5 diluted mouse anti‐RBD or the respective preimmune serum. *t*‐Test was used to compare the groups, where ****p < .0001. (D) The serum neutralization activity of each mouse is represented by the titer of inhibition of SARS‐CoV‐2 infection in Vero cells. Neutralization capacity was determined for titers >1:40 (dotted line), in different strains. Mice were immunized with PBS (control), with RBD‐multi in alum adjuvant (Alum), or with RBD‐multi and AS03 adjuvant (AS03). Statistical analysis was performed using the GraphPad Prism 9 software. Significance was determined by the two‐way analysis of variance with Tukey's multiple comparison test, where **p < .01, *<.1, and ns represents nonsignificant differences.

We used a competitive ELISA with immobilized S1 glycoprotein followed by the addition of ACE2‐biotin to see if the resulting sera from the first immunization series could block RBD binding to ACE2. In this assay, we discovered that all five alum‐immunized mice from the first round of immunization produced antibodies capable of inhibiting ACE2 binding to the viral protein, comparable to a positive control containing a neutralizing antibody (Figure 5A). ACE2‐biotin also binds to wells coated with different concentrations of the multimeric RBD adsorbed to the plate in a saturable manner (Figure 5B). Consequently, the wells were coated with 50 ng of multimeric RBD, which was the concentration in the binding assay's linear range, and then used to test for antibody inhibition when diluted at 1:5. Indeed, all sera inhibited ACE2 binding, with three of them being more effective (Figure 5C).

The sera obtained from mice after the first set of immunizations, with partially purified multimeric RBD in alum produced low neutralization titers for inhibition of viruses' infection. Hence, we compared the sera obtained by using AS03 to those with alum as adjuvant, those obtained in the second immunization set. When the multimeric RBD was injected with AS03, the resulting sera prevented Wuhan SARS‐CoV‐2 infection at concentrations greater than 1/2000. In contrast, the neutralization titers obtained by using alum were significantly lower. Importantly, the sera from mice immunized with the mutimeric RBD in AS03 also protected against infection by Delta, Gamma, and Omicron strains, although at higher concentrations. Some of the sera from mice immunized with alum prevented infection at higher concentrations in the cases of Gamma and Delta, but not of Omicron SARS‐Cov‐2.

### 3.5 Multimeric RBD induced production of IFN‐γ secreting T cells

**Figure 6:**
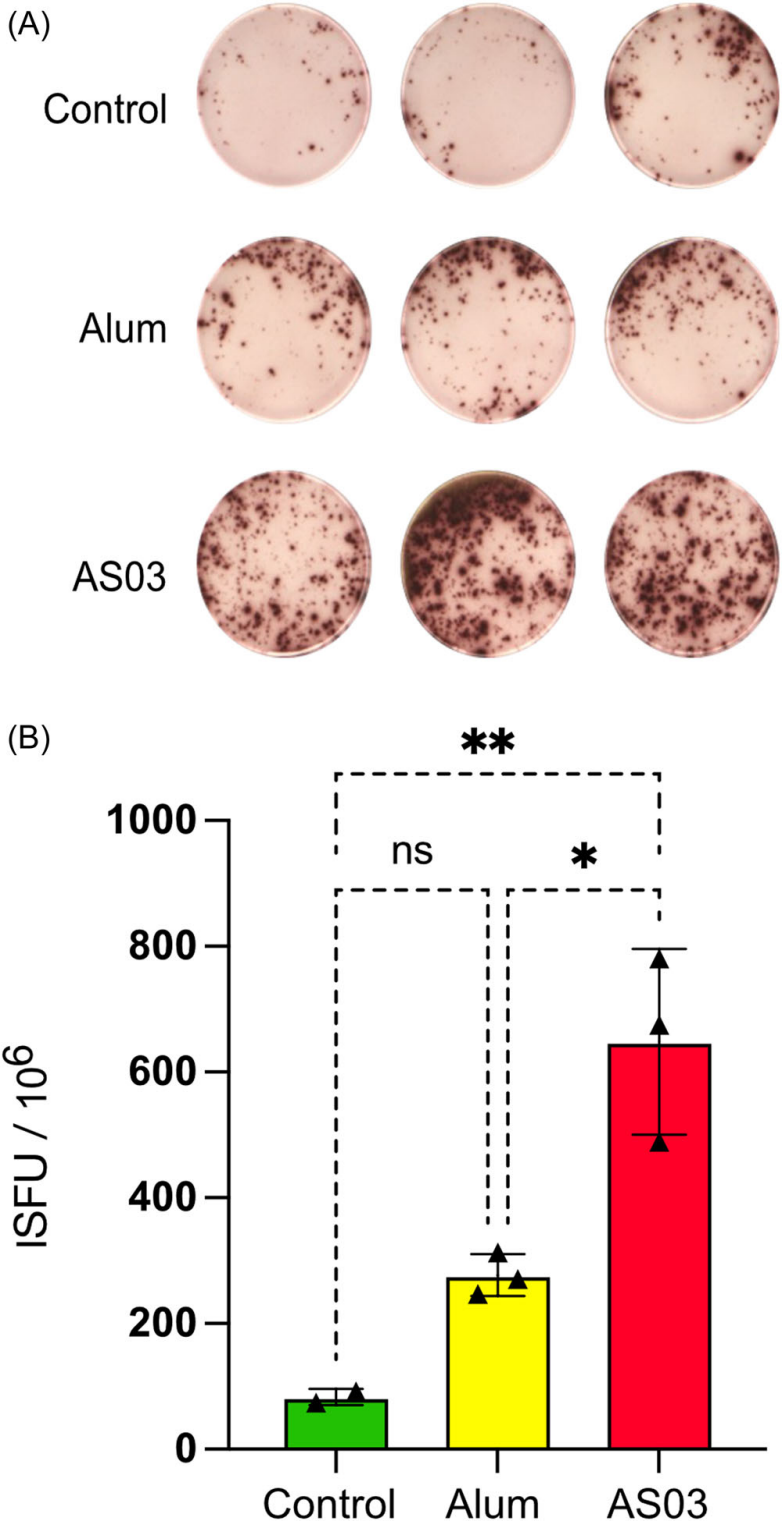
Detection of interferon (IFN‐γ)‐secreting T cells. (A) Splenocytes from mice immunized with receptor‐binding domain (RBD)‐multi, placed in triplicate in wells coated with anti‐IFN‐γ capture antibody and stimulated with RBD‐multi (5 μg/mL). Mice immunized with phosphate‐buffered saline (control), mice immunized with RBD‐multi and Alum adjuvant (Alum), and mice immunized with RBD‐multi and AS03 adjuvant (AS03). (B) Number of IFN‐γ‐secreting T cells. Statistical analysis was performed using the GraphPad Prism 9 software. Significance was determined by the one‐way analysis of variance with Tukey's multiple comparison test, where **p < .01, *<.1, and ns represents nonsignificant differences.

To determine whether immunization with multimeric RBD could induce a cellular immune response, we used splenocytes isolated from immunized mice to interact with wells coated with anti‐IFN‐γ capture antibody in a cell ELISpot assay. In comparison to control animals, splenocytes from mice immunized with multimeric RBD in AS03 and exposed to multimeric RBD presented a higher number of IFN‐ γ producing cells (Figure 6). The number of IFN‐γ producing cells was also increased in animals immunized with multimeric RBD in alum, albeit to a lesser extent.

## 4 DISCUSSION

In this work, we generated a new recombinant RBD with the full carboxy terminus of the S1 virus subunit linked to the trimerization motif of bacteriophage T4 and tested its ability to act as a protective antigen after immunization. This new recombinant protein was produced and released constitutively by HEK293 cells, which exhibited multimeric behavior, most likely trimers and hexamers. Some of the produced proteins formed multimeric aggregates of high molecular weight that could be removed using gel exclusion chromatography. Our data indicated that the trimer comprised a covalent dimer connected by an S‐S bridge, which is consistent with the presence of a cysteine in the C‐terminal region of our construct. This cysteine is located far from the ACE2 binding site, whereas other cysteines form disulfide bridges within the chain, which normally support the RBD structure.^45^ We don't know whether the trimeric or hexameric structures have the same conformation as the trimer formed by the S1 protein on the virus' surface. Nevertheless, the circular dichroism showed homogenous folding with 52.5% alpha‐helix, 18.5% beta sheets, and 5.5% coils, which is consistent with previously published spectra of monomeric RBD and supports the idea that in our case, each monomer acquires a conformation like that found in the virus. We do not know whether the formation of hexameric complexes leads to immunogenicity changes, and future experiments with known monoclonal antibodies may aid in determining best immunogen structure.

We verified that the protein immobilized in the polystyrene plate was able to be recognized by the biotin‐labeled ACE2 receptor. This recognition occurs within the RBM region located between residues 438 and 508.^46^ This region is exposed in the virus protein S1 after cleavage with furin and raised one monom the trimer to recognize the receptor.^47^ Furthermore, due to the impossibility of coupling each receptor molecule to the three subunits due to conformational impediment, only one RBD binds to ACE2 in the membrane at a time in the trimer.^48^ Some studies have shown that glycosylation is important for the interaction of RBD‐ACE2, in addition to its role in protein stability and functionality.^49^ Indeed, our protein was found to be glycosylated, as it was recognized by the lectin Concanavalin A.

Importantly, the cells generated in our study are stable and can be adapted for large‐scale growth. We also developed an ELISA/ECL assay with the recombinant multimeric RBD protein, which can be used to identify antibodies in the human population. This is because we can continuously obtain a large amount of recombinant protein without having to repeat transfection for each new purification. Furthermore, the assay could be modified to detect the presence of different classes and types of antibodies.

To evaluate the potential antigenicity of our multimeric RBD, we immunized BALB/c mice with four doses of AS03, as adjuvants. Alum, which is made up of aluminum and potassium particles, is one of the approved adjuvants for human vaccines.^50^ AS03 is a tocopherol, squalene, and polysorbate 80 oil‐water emulsion that has been successfully used to obtain protection for human immunization.^51^, ^52^, ^53^ In both cases, the mice produced specific antibodies, with AS03 generating higher titers more consistently. It appeared that AS03 elicited a faster response that stabilizes after 60 days when compared to alum, which was also able to inhibit the interaction between ACE2 and S1, or the multimeric‐RBD.

Neutralizing antibodies that target the receptor‐binding domain have been identified, with 90% neutralizing activity in convalescent sera.^54^ Early in the pandemic, studies showed that a DNA vaccine containing the RBD as well as a trimerization tag could elicit a protective immune response in a Rhesus macaque model.^20^ Because trimeric RBD interacts with ACE2 receptor molecules on the cell surface with greater affinity than monomeric RBD,^55^ it seemed reasonable to develop antibodies that recognize the trimeric structure to be more effective in inhibiting this binding. This is because immunogens with a more multimeric structure elicit a stronger humoral response in mice.^56^ Furthermore, proteins based on total protein S may have difficulty stabilizing the prefusion conformation and fail to generate adequate antibodies.^57^

The sera produced by mice immunized with AS03 were capable of neutralizing SARS‐CoV‐2 virus strain Wuhan infection in Vero cells up to a dilution of 1:2560, whereas other strains required higher concentrations. Some works have shown similar neutralization titers,^19^ while others generated higher titers using heterotrimers,^58^ large multimeric structures,^58^ or mosaic structures.^59^ In contrast, mice immunized with alum, on the other hand, produced lower neutralizing titers. The protective, but highly variable neutralizing capacity was observed for the Delta and Gamma variants, which could be explained by the presence of substitutions that reduced the affinity for critical epitopes. Lower, but consistent neutralization was found for the Omicron variant, implying the loss of some antibody binding sites.

Several studies have found cross‐reactivity among different HCoVs because they contain conserved regions.^60^ mAbs isolated before the emergence of variants demonstrated variable protective capacity.^61^ Antibodies generated by the alpha, beta, gamma, and delta variants, for example, are inefficient against Omicron, despite recognizing similar domains of the RBDs.^62^ In contrast, it has been shown that monoclonal antibodies made from a recombinant RBD (Wuhan sequence) neutralized major variants.^63^ In our case, the presence of an elongated C‐terminal domain of the S1 protein along the RBD domain may affect the production of new neutralizing antibodies. In fact, some publications suggest that the entire S1 extension is involved in the virus' interaction with the ACE2 receptor,^64^ and the generation of a strong inhibition of ACE2 binding, may imply that this portion was also involved in the generation of neutralizing antibodies in our case. This, when combined with a decrease in antibody titers after immunization, clearly demonstrates the requirement for a timely vaccine boost for the new variants.^65^ Furthermore, when SARS‐CoV‐2 binds to ACE2, its expression reduced its expression in infected cells.^66^ It would therefore be interesting to see whether the antibodies produced could also affect internalization via alternative receptors.

Immunization using RBD‐Multi and AS03 additionally induced a specific T‐cell response with a significant production of INF‐γ after stimulation with the recombinant protein RBD‐Multi. Although alum stimulated a significant IgG antibody response, it did not stimulate robust T‐cell responses. According to some studies using AS03 adjuvant, in our case, induced a stronger T cell response than alum.^67^ The use of alum in conjunction with other adjuvants may be ideal, as it has been shown in other studies to increase neutralizing antibodies when combined with CpG 1018,^68^, ^69^ which also increased T‐cell responses.^70^

Although several constructs already use trimeric RBD as an antigen, the majority of them do not use the extended (residues 331 to 589) portion of the S1 protein C‐terminal domain.^18^, ^19^, ^55^, ^71^, ^72^ The RBD domain typically expands the protein to residue 527. The presence of the expansion to residue 541 in the SARS‐CoV‐2‐CTD has been found to improve binding to ACE2 and affect the protein's immunogenicity.^73^ Because the extension (549‐589) we introduced is exposed outside the trimeric structure, we believe it may aid in the folding of RBD‐CTD and further recognition by antibodies.

As a limitation of our investigation, we only analyzed one SARS‐CoV‐2 variant at this time; further research is needed to determine whether similar results may be obtained with more current viral variants. We also employed a considerable amount of antigen in the mouse model. Reduced immunizing dosages and animal challenge would be required to determine the practicality of human vaccinations.

In conclusion, this study demonstrates that a new RBD multimeric protein can be produced and used as an efficient antigen for diagnosis and neutralization tests, as well as an alternative subunit vaccine for SARS‐CoV‐2, and a platform for the development of immunization variants. Although there are already several constructions using RBD as an antigen, this version describes an extended C‐terminal and a foldon domain generating a new multimeric form, which supports previous studies that have shown the efficacy of multimeric RBD as a SARS‐CoV‐2 vaccine. In light of the fact that we are still living in the aftermath of a pandemic and that SARS‐CoV‐2 mutations are still occurring, as well as the fact that there are no antivirals available, this work describes a tool to combat this illness, mainly due to unexpected aspects of the virus's persistence in humans.^74^, ^75^

## AUTHOR CONTRIBUTIONS

**Veronica A. de Lima**: Formal analysis; investigation; methodology; writing—original draft. **João P. S. Nunes**: Formal analysis; investigation; methodology. **Daniela S. Rosa**: Conceptualization; data curation; funding acquisition; resources; writing—review and editing. **Rodrigo Ferreira**: Formal analysis; methodology. **Maria L. V. Oliva**: Conceptualization; funding acquisition; resources. **Robert Andreata‐Santos**: Formal analysis; investigation; methodology; software. **Marcia Duarte‐Barbosa**: Formal analysis; investigation; methodology; visualization. **Luiz M. R. Janini**: Resources. **Juliana T. Maricato**: Conceptualization; data curation; investigation; methodology; supervision; writing—review and editing. **Milena A. Akamatsu**: Formal analysis; investigation; methodology. **Paulo L. Ho**: Conceptualization; data curation; formal analysis; funding acquisition; resources; supervision; writing—review and editing. **Sergio Schenkman**: Conceptualization; data curation; formal analysis; funding acquisition; project administration; resources; supervision; writing—original draft; writing—review and editing.

## CONFLICT OF INTEREST STATEMENT

The authors declare no conflict of interest.

## Supporting information

Supporting information.

## Data Availability

All data and resources described in this manuscript are available upon request.
