# Supplementary material for: Development and characterization of a multimeric recombinant protein using the spike protein receptor binding domain as an antigen to induce SARS‐CoV‐2 neutralization
Source: Immun Inflamm Dis. 2024 Jul 26;12(7):e1353. doi: 10.1002/iid3.1353 (PMC11273545; doi:10.1002/iid3.1353)
Supplement: Supplementary file 1 — Supporting information. [file IID3-12-e1353-s001.pdf]

Figure S1. Sequence inserted into the pcDNA3.1 plasmid.

5’ATGGAGACAGACACACTCCTGCTATGGGTACTGCTGCTCTGGGTTCCAGGTTCCACTGGTGA
CGCGGCCCAGCCGGCCAGGCGCGCCGTACGAAGCTTGAACATCACTAACTTGTGCCCATTTGGG
GAGGTCTTCAACGCAACCCGGTTCGCCTCTGTCTACGCATGGAATAGAAAAAGGATCAGCAACT
GCGTCGCAGACTATAGCGTGCTGTATAACAGTGCCAGTTTCTCAACGTTTAAGTGCTACGGGGT
GAGCCCAACCAAGCTGAATGACCTCTGCTTTACAAATGTGTACGCAGATTCTTTTGTGATTAGA
GGAGACGAGGTCCGGCAAATCGCACCGGGCCAGACAGGCAAAATAGCTGACTACAACTACAAGC
TCCCGGATGACTTCACAGGGTGCGTGATAGCTTGGAATAGCAATAACCTTGACTCTAAAGTGGG
GGGAAATTACAACTATCTGTACCGACTGTTCAGAAAGTCAAACCTCAAGCCCTTCGAGCGCGAT
ATTTCAACTGAGATCTATCAAGCTGGGTCTACCCCTTGCAATGGCGTGGAAGGATTCAATTGTT
ACTTCCCCCTGCAGTCCTACGGATTCCAGCCCACTAACGGCGTGGGGTATCAACCTTATCGGGT
GGTCGTGCTCAGCTTTGAGCTTCTTCACGCCCCAGCGACCGTCTGCGGCCCCAAGAAGTCCACC
AACCTGGTGAAGAACAAGTGCGTGAACTTCAACTTCAACGGCCTGACCGGCACCGGCGTGCTGA
CCGAGTCCAACAAGAAGTTCCTGCCCTTCCAGCAGTTCGGCCGCGACATCGCCGACACCACCGA
CGCCGTGCGCGACCCCCAGACCCTGGAGATCCTGGACATCACCCCCGGCTCCGGCTACATCCCC
GAGGCCCCCCGCGACGGCCAGGCCTACGTGCGCAAGGACGGCGAGTGGGTGCTGCTGTCCACCT
TCCTGTCTCCGGGTAAACTCGAGGGTACCGGCGGCAGCCACCATCATCATCACCAT’3
